# Protective Role of the MER Tyrosine Kinase *via* Efferocytosis in Rheumatoid Arthritis Models

**DOI:** 10.3389/fimmu.2018.00742

**Published:** 2018-04-13

**Authors:** Claire E. J. Waterborg, Silke Beermann, Mathijs G. A. Broeren, Miranda B. Bennink, Marije I. Koenders, Peter L. E. M. van Lent, Wim B. van den Berg, Peter M. van der Kraan, Fons A. J. van de Loo

**Affiliations:** Experimental Rheumatology, Department of Rheumatology, Radboud Institute for Molecular Life Sciences, Radboud University Medical Center, Nijmegen, Netherlands

**Keywords:** rheumatoid arthritis, experimental arthritis, MER tyrosine kinase, efferocytosis, anti-inflammatory

## Abstract

**Objective:**

Rheumatoid arthritis (RA) is a chronic and progressive joint disease. It appears that anti-inflammatory feedback mechanisms that could restrain joint inflammation and restore homeostasis are insufficient to perform this control. In this study, we investigated the contribution of the MER tyrosine kinase-mediated anti-inflammatory response on arthritis and whether targeting MER could be a valid approach to treat RA.

**Methods:**

KRN serum transfer arthritis (KRN STA) was induced in either *Mertk*-deficient mice or in mice that adenovirally overexpressed *Pros1*. Human synovial micromasses were treated with MER-specific antibodies or PROS1. Collagen-induced arthritis (CIA) mice were treated with MER-specific agonistic antibodies or by viral overexpression of *Pros1*.

**Results:**

*Mertk^−/−^* mice showed exacerbated arthritis pathology, whereas *Pros1* overexpression diminished joint pathology in KRN STA. Human synovial micromasses challenged with MER-specific antibodies enhanced the secretion of inflammatory cytokines, whereas stimulating MER with PROS1 reduced the secretion of these cytokines, confirming the protective role of MER. Next, we treated CIA mice with MER-specific agonistic antibodies, and this unexpectedly resulted in exacerbated arthritis pathology. This was associated with increased numbers of apoptotic cells in their knee joints and higher serum levels of interleukin (IL)-16C, a cytokine released by secondary necrotic neutrophils. Apoptotic cell numbers and IL-16C levels were enhanced during arthritis in *Mertk^−/−^* mice and reduced in *Pros1*-overexpressing mice.

**Conclusion:**

MER plays a protective role during joint inflammation and activating MER by its ligand PROS1 ameliorates disease. Treatment of mice with MER receptor agonistic antibodies is deleterious due to its counterproductive effect of blocking efferocytosis in the arthritic joint.

## Introduction

Rheumatoid arthritis (RA) is a chronic disease that is characterized by synovitis and damage of both cartilage and bone in synovial joints, leading to functional disabilities that affect up to 1% of the population worldwide (1, 2). The current generation of biological drugs are inhibiting inflammatory cytokines, such as tumor necrosis factor alpha (TNF-α), interleukin (IL)-6, and IL-1, depleting B cells by targeting cluster of differentiation (CD) 20 or by inhibiting the co-stimulation of T cells by antigen-presenting cells (3). Although biologicals have increased the treatment efficacy of RA patients, a substantial percentage of RA patients do not respond or are refractory to current treatment. Furthermore, side effects of biologicals are increased risk of infections and the development of other autoimmune diseases (4). Restoring homeostasis by for example the administration of the anti-inflammatory cytokine IL-10 has shown little efficacy in clinical trials either due to counteractive effects or poor pharmacokinetics of this protein (5, 6). Activation of natural negative feedback mechanisms as a potential therapy for RA has not been fully explored.

The TAM receptors—TYRO3, AXL, and MER (gene name *MERTK*)—are receptor tyrosine kinases that play a critical role in natural anti-inflammatory feedback mechanisms. The two principal TAM receptor protein ligands are growth arrest-specific 6 (GAS6) and Protein S (PROS1). Notably, GAS6 is a ligand for all three receptors but with the highest affinity for AXL. In contrast, PROS1 can only activate TYRO3 and MER, but not AXL (7). These ligands display divalent binding activities: they bind to TAM receptors through a carboxy-terminal domain, and also, *via* an amino-terminal domain, to phosphatidylserine (PS) that is expressed as an “eat-me signal” on the surface of apoptotic cells (7). PROS1/GAS6 binding to PS effectively opsonizes apoptotic cells for TAM receptor-mediated phagocytic uptake, a process called efferocytosis (8, 9). Additionally, the TAM receptors negatively regulate inflammation, among others by inducing Suppressor Of Cytokine Signaling (SOCS) proteins 1 and 3 (10–16). SOCS1 and 3 inhibit TLR- and cytokine receptor signaling, resulting in reduced production of pro-inflammatory cytokines (10, 16, 17). The TAM receptors can also be shed from the cell surface thereby creating a soluble ectodomain. For MER, the enzyme responsible for this shedding is A Disintegrin AND Metallopeptidase Domain 17 (ADAM17) (18). By competing for the ligands with the membrane-bound MER, soluble MER has been shown to inhibit efferocytosis (19–21).

TAM receptors have been associated with numerous inflammatory diseases, such as multiple sclerosis, atherosclerosis, and various rheumatic diseases (22–26). These studies focused mainly on the association of the soluble ectodomains of the TAM receptors with disease activity parameters. We have previously shown that both systemic and intra-articular adenoviral overexpression of *Gas6* and *Pros1* in collagen-induced arthritis (CIA) reduces inflammation and bone and cartilage erosion in murine knee joints (16). The objective of this study was to illuminate the endogenous role of the MER tyrosine kinase, and its role upon PROS1 stimulation, in two different experimental models of arthritis and a three-dimensional model of the human synovium.

## Materials and Methods

### Antibodies

The list of antibodies, origin, and function are given in Table 1.

**Table 1:** List of antibodies, origin, and function.

Antibody	Reactivity	Company	Catalog number	Monoclonal/polyclonal	Function	Figure (F)/Supplementary Figure (SF)
MER	Human	Abcam	ab52968	Monoclonal [Y323]	Immunohistochemistry	F3A
IgG for MER	–	DAKO	X0936	–	Immunohistochemistry	F3A
CD68	Human	DAKO	M0814	Monoclonal [KP1]	Immunohistochemistry	F3A
IgG for CD68	–	DAKO	X0931	–	Immunohistochemistry	F3A
Secondary antibody	Mouse	DAKO	P0260	Polyclonal	Immunohistochemistry	F3A
MER	Human	Abcam	ab176887	Monoclonal [Y323]	Neutralization	F3BSF3A,B,C
IgG for MER	–	Abcam	ab176094	–	Neutralization	F3BSF3A,B,C
MER	Mouse	R&D systems	AF591	Polyclonal	Activation	F4F5A,BF6A,B,C,DSF4, SF5
IgG for MER	–	R&D systems	AB-108-C	–	Activation	F4F5A,BF6A,B,C,DSF4, SF5
MER	Mouse	R&D systems	AF591	Polyclonal	Immunoprecipitation	F4A
MER	Mouse	R&D systems	AF591	Polyclonal	Immunoblotting	F4A
Phosphorylation	Mouse	Millipore	05-321	Monoclonal [4G10]	Immunoblotting	F4A
Cleaved Caspase-3	Mouse	BD Pharmingen	559565	Monoclonal [C92-605]	Immunohistochemistry	F5
IgG for Cleaved Caspase-3	–	DAKO	X0936	–	Immunohistochemistry	F5
MER	Mouse	eBioscience	12-5751	Monoclonal [DS5MMER]	Flow cytometry	SF1A
F4/80	Mouse	Bio-Rad	MCA497	Monoclonal [Cl:A3-1]	Immunofluorescence	F6ASF5A
Secondary antibody	Rat	Molecular probes	A11006	Polyclonal	Immunofluorescence	F6ASF5A

### *In Vivo* 

#### Mice

Male DBA/1 (Janvier) were used for the CIA model and housed in filter top cages. Male C57BL/6 mice (Janvier) were used for KRN serum transfer arthritis (STA) experiments using viral overexpression in knee joints. Mice were housed in individually ventilated cages. Both mouse strains were used at an age range of 10–12 weeks. The *Mertk^−/−^* strain was generated as described previously (27). All lines were backcrossed for >9 generations to a C57BL/6 background. Male *Mertk^−/−^* mice and wild-type (WT) littermates at 10 weeks of age were used for CIA and KRN STA experiments and housed in individually ventilated cages. Male and female mice on a C57BL/6 background were used for bone marrow isolations. All mice were fed a standard diet with freely available food and water. Mice which received a treatment (adenovirus or antibody) were randomly allocated to experimental groups. Histological and immunohistochemical analyses were performed in a randomized and blinded manner. Clinical signs of arthritis in paws and ankle joints were monitored macroscopically three times per week. Cumulative scoring was based on redness, swelling, and, in later stages, ankylosis, with a maximal score of 2 per paw. Humane endpoint was defined as reaching an individual score higher than 6 (on a scale of 0–8), followed by euthanization of the mouse. All *in vivo* studies performed in The Netherlands complied with Dutch legislation and were approved by local authorities for the care and use of animals with related codes of practice. The *in vivo* studies executed in The United States of America were conducted according to guidelines established by the Salk Institutional Animal Care and Use Committee. Group sizes were determined by power calculation on basis of incidence, mean, and SD, and are indicated per experiment.

#### KRN STA

KRN STA was induced by two intraperitoneal injections, at day 0 and 2, of 150 µL arthritic K/BxN serum in either WT or *Mertk^−/−^* mice or in WT C57BL/6J mice that virally overexpressed *luciferase* (Ad Luc) or *Pros1* (Ad Pros1) in their knee joints. The overexpression of *luciferase* or *Pros1* was accomplished by an intra-articular injection into the knee joint of 1 × 10^7^ plaque-forming units (PFU) of adenovirus, 24 h prior to the first serum injection. Mice were euthanized at day 7 or 14, respectively.

#### Collagen-Induced Arthritis

For induction of CIA in DBA/1 mice, bovine type II collagen was dissolved in 0.05 M acetic acid at a concentration of 2 mg/mL and emulsified in an equal volume of Freund’s complete adjuvant (2 mg/mL of *Mycobacterium tuberculosis* strain H37Ra; Difco). Mice were immunized with 100 µL of this emulsion intradermally at the base of the tail. At day 21, mice were given an intraperitoneal booster injection of 100 µg of type II collagen dissolved in phosphate-buffered saline (PBS). One day after the booster injection, mice were injected intravenously with 10 µg of anti-MER (AF591) or IgG (AB-108-C) or 3 × 10^8^ PFU of Ad Luc or Ad Pros1. Mice were euthanized at day 30 or day 36, respectively.

### *In Vitro*—Human

#### Patient Material

RA synovial tissue was obtained during surgery from the Radboud University Medical Center or the Sint Maartenskliniek (both the Netherlands). This material was considered surgery surplus material. For the Radboud University Medical Center, patients gave informed consent for the surgery, were informed and were able to decline the use of their material for research. According to Dutch law, informed consent was not necessary. In addition, patient material was anonymized. For the Sint Maartenskliniek, patients gave written informed consent for the use of their material for research. The patient material was pseudonymized. Therefore, there was no need for the approval by an ethical committee. Protocols were performed in accordance to the code of conduct for responsible use of human tissue in medical research (Gedragscode 2011, https://www.federa.org/code-goed-gebruik). Synovial samples were digested using Liberase TM (50 µg/mL; Roche) for 2 h at 37°C in Roswell Park Memorial Institute (RPMI) medium (Gibco). Subsequently, red blood cells were lysed. Cells were put in culture with RPMI medium supplemented with 10% heat-inactivated fetal calf serum (FCS), 1 mM pyruvate and penicillin/streptomycin (P/S). Medium was refreshed weekly. Cells were kept at 37°C with 5% CO_2_.

#### Human Monocytes

Whole blood from healthy donors was mixed with PBS 1.5% acid citrate-dextrose solution A 1:1. Peripheral blood mononuclear cells were isolated by a density gradient centrifugation method using Ficoll-Paque PLUS (GE Healthcare). CD14^+^ cells were isolated with the MagniSort Human CD14 Positive Selection Kit according to manufacturer’s protocol (Invitrogen) (purity >90%).

#### Human Synovial Three-Dimensional Micromasses

For the construction of micromasses, rheumatoid arthritis fibroblast-like synoviocytes (RAFLS) obtained from synovial samples (see Patient Material) and monocytes were mixed with a ratio of 1:5 (200.000 RAFLS and 1 × 10^6^ monocytes per micromass). The cell suspension was centrifuged and cell pellets were dissolved in ice-cold Matrigel (Corning). Using cooled pipette tips, 25 µL droplets were placed in 24-well culture plates which were coated with poly-(2-hydroxyethyl methacrylate) (Sigma-Aldrich). After 30 min gelation at 37°C, RPMI medium supplemented with 10% FCS, 1 mM pyruvate and P/S was added. Micromasses were kept at 37°C with 5% CO_2_. After 7 days of culture, the formation of a synovial-like lining was confirmed on histology (not shown). Subsequently, experiments were set in. In one set of experiments, micromasses were pre-incubated with 50 nM PROS1 (Haematologic Technologies) for 24 h before stimulation with 100 ng/mL *E. coli* lipopolysaccharide (LPS) (Invivogen), 100 ng/mL Pam3Cys-Ser(Lys)4 (P3C) (Invivogen) or 10 ng/mL recombinant human TNF-α (Abcam). After 6 h, cells were lysed in TRIzol (Sigma-Aldrich) and processed for quantitative PCR analysis. Supernatants were collected after 24 h for further analysis. In another set of experiments, micromasses were treated with human anti-MER [Abcam; as previously described (28)]. Supernatants were collected after 24 h for further analysis.

### *In vitro*—Murine

#### *In Vitro Pros1* Overexpression

Bone marrow cells were isolated by flushing murine femur and tibia bones followed by red blood cell lysis. Cells were differentiated into bone marrow-derived macrophages (BMMs) by culturing them in the presence of 15 ng/mL M-CSF (R&D). Medium was refreshed at day 4. At day 7, BMMs were transduced with adenoviruses encoding for *luciferase* or *Pros1* with a multiplicity of infection of 200. Viruses were removed after 2 h. After 48 h, cells were stimulated with 100 ng/mL LPS or 100 ng/mL P3C. After 6 h, cells were lysed in TRIzol and processed for quantitative PCR analysis. Supernatants were collected after 24 h for further analysis.

#### Cell Lines

J774A.1 macrophages were maintained in Dulbecco’s modified eagle medium (Life Technologies). EL-4 T cells were cultured in RPMI medium. Both media were supplemented with 10% FCS, 1 mM pyruvate and P/S. Cells were kept at 37°C with 5% CO_2_.

#### Apoptosis Induction

EL-4 cells were serum-starved for 16 h and incubated with 2 µM Staurosporine for 4 h. This resulted in 70% apoptotic cells verified by Annexin V and 7-AAD staining and flow cytometry, measured on FACSCalibur (BD Biosciences).

#### *In Vitro* Efferocytosis Assay

J774A.1 cells were cultured on eight-well chamber slides, serum-starved for 16 h, and pre-incubated with anti-MER or IgG for 30 min. Apoptotic cells were labeled with pHrodo (Life Technologies) as described previously (29). J774A.1 cells were co-incubated with a 10-fold excess of apoptotic cells in presence of anti-MER or IgG for 2 h. Excess of apoptotic cells was removed and J774A.1 cells were fixed with 4% paraformaldehyde (PFA). Slides were incubated with rat anti-mouse F4/80 (Bio-Rad) followed by AF488-labeled goat anti-rat IgG (Invitrogen). Nuclei were stained with DAPI. Pictures were taken and the ratio of phagocytic macrophages compared to total number of macrophages was determined per well. Cells were counted as phagocytic if there was at least one pHrodo-labeled apoptotic cell within the F4/80-labeled membrane. The quantification was performed in a randomized and blinded manner by two independent observers.

#### *In Vitro* Secondary Necrosis Assay

J774A.1 cells were cultured on eight-well chamber slides. Bone marrow neutrophils were isolated using Anti-Ly-6B.2 (7/4) Micro-Beads (Miltenyi Biotec) according to manufacturer’s protocol after flushing tibia and femur bones and red blood cell lysis. Neutrophils were cocultured with J774A.1 cells at a ratio 2:1 in the presence of 5 µg/mL anti-MER or IgG for 24 or 48 h. For microscopy, cells were stained with F4/80 and DAPI as described for “*in vitro* efferocytosis assay” and representative pictures were taken. Supernatants after 48 h coculture were collected for further analysis.

### Other

#### Adenoviruses

First generation adenoviruses encoding for *luciferase* (Ad Luc) and *Pros1* (Ad Pros1) were produced as previously described (16).

#### Flow Cytometry MER

Bone marrow-derived macrophages were incubated with mouse Fc Block (BD Pharmingen) to block Fcγ receptors, diluted in FACS buffer [PBS, 5% FCS, 2 mM ethylenediaminetetraacetic acid (EDTA)]. Subsequently, surface marker expression was evaluated using rat anti-mouse MER PE (eBioscience). Samples were measured on BD FACSCalibur (BD Biosciences) and analyzed with FlowJo software (Tree Star).

#### Histological and Immunohistochemical Analysis

Micromasses at day 7 of culture were fixed for 2 h in 2% PFA in PBS/1 mM CaCl_2_, dehydrated, and embedded in paraffin. Sections were deparaffinized, rehydrated, and stained with hematoxylin and eosin to confirm synovial-like lining formation or were further processes for immunohistochemistry. Knee joints were fixed in formalin, decalcified with formic acid, and embedded in paraffin. Tissue sections were stained with safranin O and fast green (both BHD Chemicals) or were further processed for immunohistochemistry. Three semi-serial sections per joint were scored for histopathologic changes on an arbitrary scale from 0 to 3, by two independent observers in a blinded manner as described previously (30). The average of the three semi-serial sections is depicted per joint. Joint inflammation was determined by the presence of synovial cell infiltrates and inflammatory cell exudates. Connective tissue destruction was determined by the depletion of proteoglycans and by cartilage and bone erosion. CD 68 and MER were evaluated on paraffin section of micromasses. Cleaved Caspase-3 was evaluated on paraffin sections of murine knee joints. Sections were deparaffinized and rehydrated. Antigen-retrieval was performed in Tris/EDTA buffer at 60°C for MER and citrate buffer at 60°C for CD68 and cleaved Caspase-3. Endogenous peroxidase was blocked by 3% hydrogen peroxide. Tissue sections were incubated with 0.2 µg/mL rabbit anti-human MER (Abcam) or control rabbit IgG followed by incubation with biotinylated goat anti-rabbit (Vector Labortatories), 41 µg/mL mouse anti-human CD68 (DAKO), or control mouse IgG, followed by rabbit anti-mouse horseradisch peroxidase-labeled antibody (DAKO), or 0.5 µg/mL rabbit anti-cleaved Caspase-3 (BD Bioscience) or control rabbit IgG followed by incubation with biotinylated goat anti-rabbit (Vector Labortatories). A biotin–streptavidin detection system was used according to manufacturer’s protocol for MER and cleaved Caspase-3 (Vector Laboratories). Bound complexes were visualized *via* reaction with diaminobenzidine (Sigma-Aldrich). All sections were counterstained with hematoxylin. For cleaved Caspase-3, two to six pictures were taken of different areas of each joint section in a standardized and blinded manner. Three semi-serial tissues sections per joint were analyzed. The average of the three semi-serial sections was depicted per joint. Quantification of the staining was performed using the LAS V4.3 software (Leica). The inflamed area was selected and the area of cleaved Caspase-3 positive cells was expressed as percentage.

#### RNA Isolation and Quantitative PCR Analysis

3-mm synovial biopsies were disrupted using the MagNA Lyser (Roche). Biopsies, BMMs and micromasses were lysed with TRIzol. Total RNA was extracted using TRIzol/Chloroform extraction and treated with DNAse followed by reverse transcription into cDNA using oligo(dT) primers. Quantitative PCR was performed with SYBR green PCR master mix using the StepOnePlus Real-Time PCR System (Applied Biosystems). Glyceraldehyde-3-phosphate dehydrogenase (*Gapdh* or *GAPDH*) was used as reference gene. The primer sequences are listed in Table 2. The C_T_ value was set to a threshold of 40 in the samples in which no C_T_ value was detected.

**Table 2:** List of oligonucleotide primer sequences.

Species	Oligo description	Sequence (5′ → 3′)
Human	GAPDH_FW	ATCTTCTTTTGCGTCGCCAG
Human	GAPDH_RV	TTCCCCATGGTGTCTGAGC
Human	IL1B_FW	TGGGTAATTTTTGGGATCTACACTCT
Human	IL1B_RV	AATCTGTACCTGTCCTGCGTGTT
Human	TNF_FW	TCTTCTCGAACCCCGAGTGA
Human	TNF_RV	CCTCTGATGGCACCACCAG
Human	ADAM17_FW	TCACGTTTGCAGTCTCCAAA
Human	ADAM17_RV	CACTCGATGAACAAGCTCTTC
Human	PROS1_FW	CCCTGGAGGTTACACTTGCT
Human	PROS1_RV	ACTGCTCCGCCAAGTAAAGT
Mouse	Gapdh_FW	GGCAAATTCAACGGCACA
Mouse	Gapdh_RV	GTTAGTGGGGTCTCGCTCCTG
Mouse	Il1b_FW	GGACAGAATATCAACCAACAAGTGATA
Mouse	Il1b_RV	GTGTGCCGTCTTTCATTACACAG
Mouse	Tnf_FW	CAGACCCTCACACTCAGATCATCT
Mouse	Tnf_RV	CCTCCACTTGGTGGTTTGCTA
Mouse	Ccl2_FW	TTGGCTCAGCCAGATGCA
Mouse	Ccl2_RV	CCTACTCATTGGGATCATCTTGCT
Mouse	Ccl3_FW	TTGGCTCAGCCAGATGCA
Mouse	Ccl3_RV	CCTACTCATTGGGATCATCTTGCT
Mouse	Mmp3_FW	TGAAGCCACCAACATCAGGA
Mouse	Mmp3_RV	TGGAGCTGATGCATAAGCCC
Mouse	Mmp13_FW	AGACCTTGTGTTTGCAGAGCACTAC
Mouse	Mmp13_RV	CTTCAGGATTCCCGCAAGAG
Mouse	Mmp14_FW	GCCTGCATCCATCAAACT
Mouse	Mmp14_RV	CAGTGCTTATCTCCTTTGAAGAAG
Mouse	Il17a_FW	CAGGACGCGCAAACATGA
Mouse	Il17a_RV	GCAACAGCATCAGAGACACAGAT
Mouse	Il6_FW	CAAGTCGGAGGCTTAATTACACATG
Mouse	Il6_RV	ATTGCCATTGCACAACTCTTTTCT
Mouse	Cxcl1_FW	TGGCTGGGATTCACCTCAA
Mouse	Cxcl1_RV	GAGTGTGGCTATGACTTCGGTTT
Mouse	Mmp9_FW	GGAACTCACACGACATCTTCCA
Mouse	Mmp9_RV	GAAACTCACACGCCAGAAGAATTT
Mouse	Socs1_FW	CCGTGGGTCGCGAGAAC
Mouse	Socs1_RV	AAGGAACTCAGGTAGTCACGGAGTA
Mouse	Socs3_FW	TAGACTTCACGGCTGCCAAC
Mouse	Socs3_RV	CGGGGAGCTAGTCCCGAA
Mouse	Pros1_FW	GCACAGTGCCCTTTGCCT
Mouse	Pros1_RV	CAAATACCACAATATCCTGAGACGTT
Mouse	Adam17_FW	ATCGTTGGGTCTGTTCTGGTT
Mouse	Adam17_RV	ATCTCAATGTTACTGTGATGAAACA

#### Measurement of Cytokines and Chemokines

Cytokines and chemokines in sera and supernatants were measured on a Bio-Plex 200 system using a magnetic bead-based multiplex immunoassay. Data analysis was performed with Bio-Plex Manager software (all Bio-Rad). IL-16C in sera and super-natants was examined using the mouse IL-16 DuoSet ELISA (DY1727; R&D systems). Murine soluble MER in sera and supernatants was examined using the mouse MER DuoSet ELISA (DY591; R&D systems). Human soluble MER in supernatants was examined using the total human MER DuoSet ELISA (DYC891; R&D systems). All ELISAs of R&D systems were used according to manufacturer’s instructions using the DuoSet Ancillary Reagent kit 2 (DY008; R&D systems).

#### Immunoprecipitation and Immunoblotting

Tissues were snap frozen in liquid nitrogen and processed for immunoprecipitation and immunoblotting as described previously (31). Tissues lysed on ice in lysis buffer. Cell lysates were incubated overnight at 4°C with protein-G/A Sepharose to pre-clear lysates. For immunopreciptation (IP), cell lysates were incubated for 2.5 h at 4°C with anti-Mer. Protein G-Sepharose (Invitrogen) was added for 2 h and immunoprecipitates (IPs) were washed twice with lysis buffer containing 0.5 M NaCl and once with 50 mM Tris/HCl pH 7.5. IPs were eluted in SDS sample buffer, separated on polyacrylamide gels, and transferred to PVDF membranes. Membranes were blocked in TBST containing 5% bovine serum albumin and immunoblotted overnight at 4°C with primary antibodies diluted 1:1,000 in blocking buffer. Blots were then washed in TBST and incubated for 1 h at RT with secondary HRP-conjugated antibodies (GE Healthcare) diluted 1:5,000 in 5% skim milk in TBST. After repeating the washes, signal was detected with enhanced chemiluminescence reagent.

### Statistics

Data are depicted as dot-plots, dot-plots with mean, mean + SD, or mean + SEM, as indicated per figure. Analysis of significance was performed using a two-tailed paired or unpaired *t*-test as indicated per experiment when comparing two groups. One-way ANOVA with Bonferroni’s multiple comparison test was used if more than two groups were compared. All data were analyzed with GraphPad Prism 5. Number of samples is depicted per experiment in figure legends. *p*-Values equal to or lower than 0.05 were considered to be statistically significant.

## Results

### Genetic Ablation of *Mertk* Results in Enhanced Arthritis Severity, Both Clinically and Histologically

**Figure 1:**
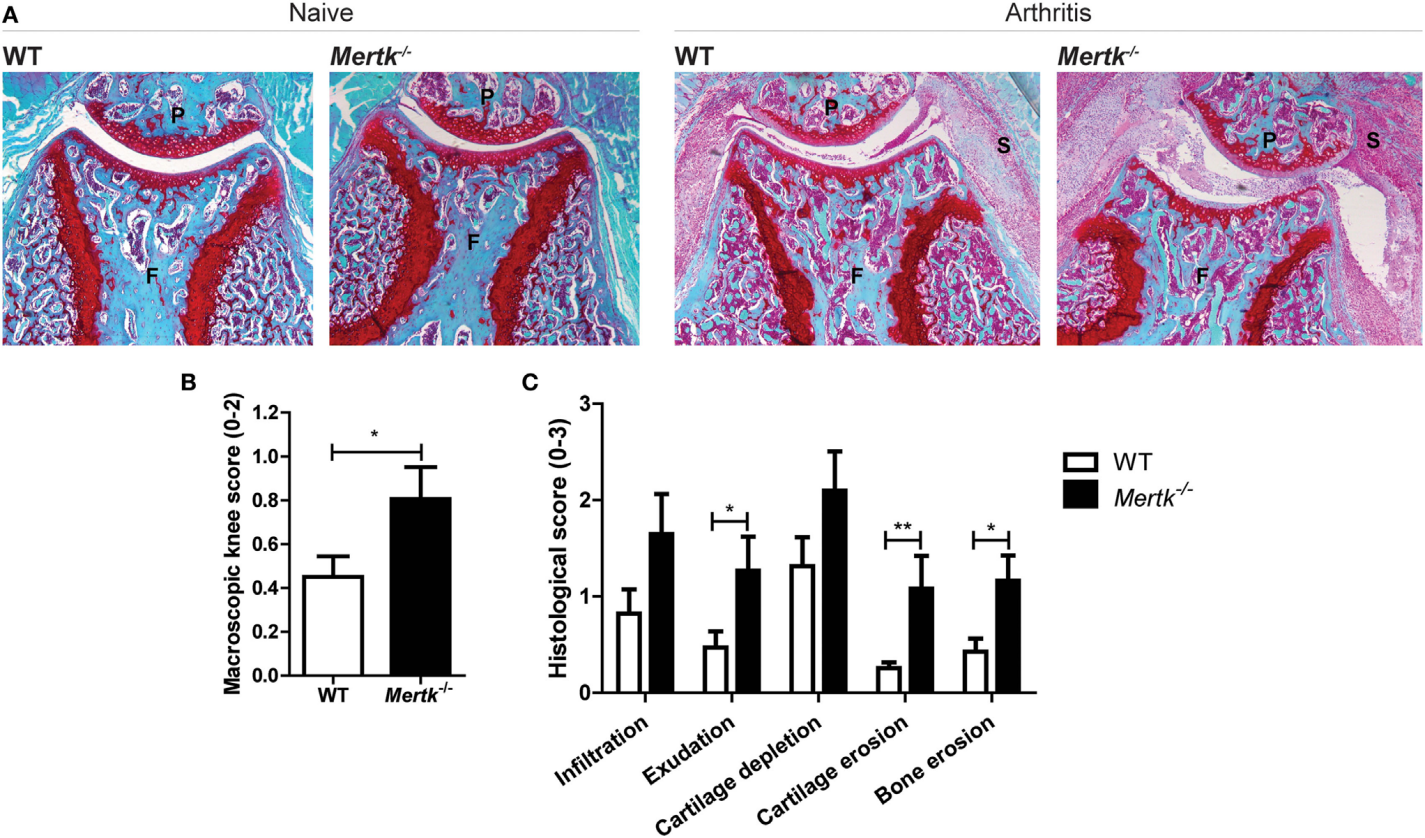
Effect of genetic ablation of *Mertk* on naive and arthritic knee joints with KRN serum transfer arthritis (STA). **(A)** Naive (*n* = 6 mice) or arthritic (KRN STA) wild-type (WT) and *Mertk^−/−^* mice (*n* = 9–15 mice) were euthanized at day 7. Shown are representative pictures of histological knee joint sections stained with safranin O and fast green. **(B)** Macroscopic score of knee joints of arthritic mice as determined by swelling and blood vessel formation (*n* = 18–30 knee joints). **(C)** Histological knee joint sections of arthritic mice were scored for the arthritis parameters depicted. Three semi-serial sections per joint were scored in a random and blinded manner with an arbitrary score from 0 to 3 (*n* = 10–18 knee joints). P, patella; F, femur; S, synovium. For **(B,C)**, data are represented as mean + SEM. *p < 0.05, **p < 0.01 with unpaired *t*-test.

Naive *Mertk^−/−^* mice did not develop arthritis spontaneously in their knee joints and these knee joints were indistinguishable from WT knee joints (Figure 1A). KRN STA was induced and 7 days later, more severe joint inflammation both clinically (Figure 1B) as well as histologically (Figures 1A,C) in *Mertk^−/−^* as compared to WT mice was observed. In addition, knee joints of *Mertk^−/−^* demonstrated more cartilage proteoglycan depletion, and cartilage and bone erosion than WT mice (Figures 1A,C).

### Adenoviral Overexpression of *Pros1* Reduces Inflammatory and Destructive Mediators and Arthritis Pathology

**Figure 2:**
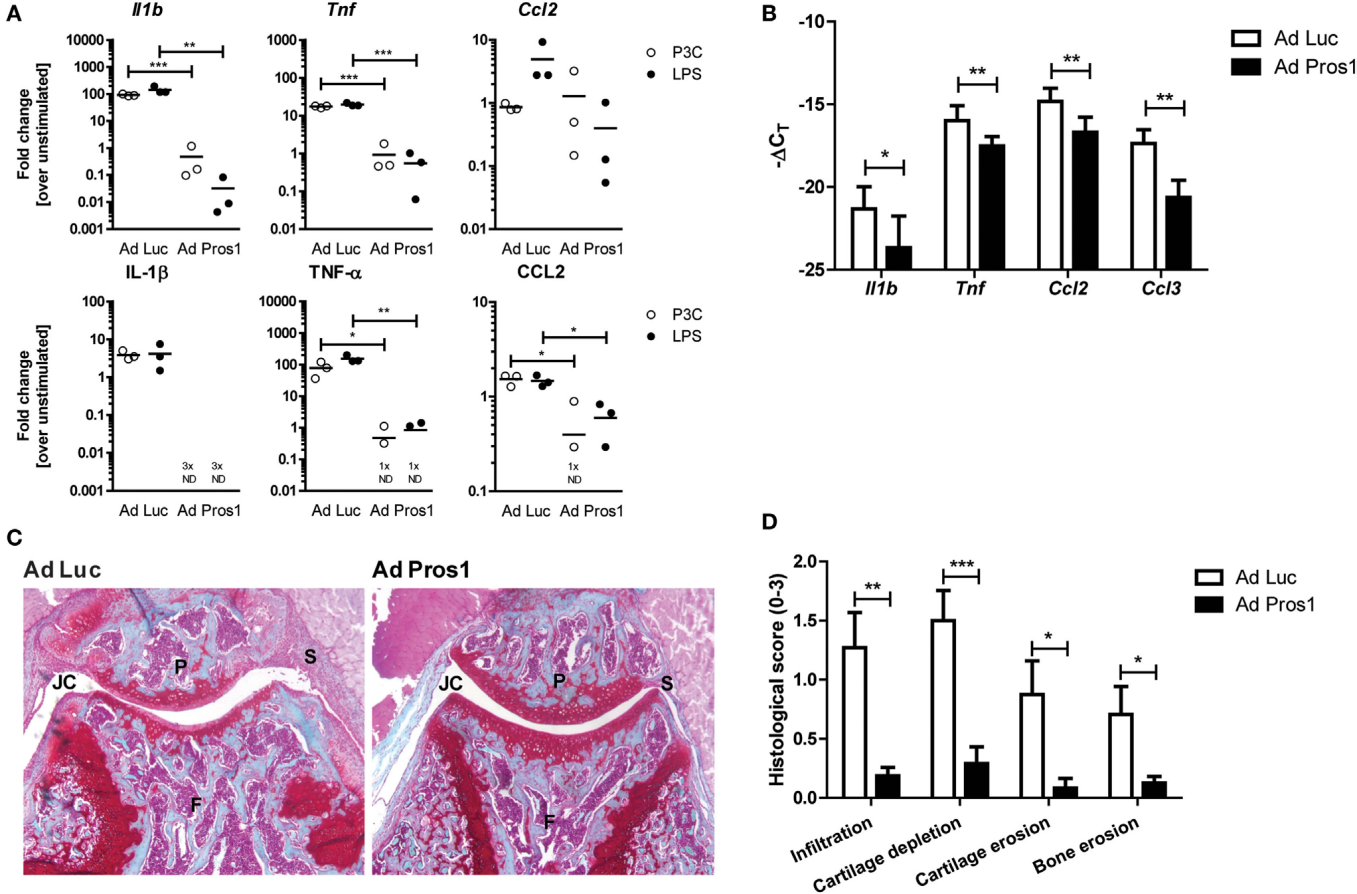
Outcome on inflammation and arthritis pathology following adenovirally overexpressing *Pros1* in KRN serum transfer arthritis (STA). **(A)** Bone marrow-derived macrophages were transduced with Ad Luc or Ad Pros1 and stimulated with lipopolysaccharide (LPS) or P3C for 6 (top) or 24 h (bottom). Messenger RNA was extracted and gene expression was determined (top). Supernatants were analyzed (bottom). Data are representative for two independent experiments (*n* = 3 per experiment). **(B)** KRN STA was induced in mice intra-articularly injected with Ad Luc or Ad Pros1 (*n* = 9 mice). Knee synovial biopsies were obtained, mRNA was extracted and gene expression was determined (*n* = 6 knee synovial biopsies). **(C)** Shown are representative pictures of knee joint sections stained with safranin O and fast green. **(D)** Histological knee joint sections were scored for the arthritis parameters depicted. Three semi-serial sections per joint were scored in a random and blinded manner with an arbitrary score from 0 to 3 (*n* = 12 knee joints). P, patella; F, femur; S, synovium; JC, joint cavity. For **(A)**, data are presented as dot-plots with mean. For **(B–D)**, data are represented as mean + SEM. *p < 0.05, **p < 0.01, ***p < 0.001 with unpaired *t*-test. See also Figures S1 and S2 in Supplementary Material.

*Mertk* deficiency showed an endogenous protective role of MER during arthritis. Therefore, next, we tested whether MER activation by PROS1 could further enhance this protection. Murine BMMs were transduced with an adenovirus overexpressing *Luciferase* (Ad Luc) or *Pros1* (Ad Pros1) 48 h prior to activation of TLR4 and TLR2. BMMs expressed prominent levels of MER and adenoviral overexpression of *Pros1* resulted in enhanced expression of *Pros1* (Figures S1A,B in Supplementary Material). *Pros1* overexpression almost completely abolished the LPS- and P3C-induced gene and protein expression of IL-1β and TNF-α (Figure 2A). In addition, CCL2 protein, but not mRNA levels, were significantly reduced by Ad Pros1 (Figure 2A). Moreover, *Adam17* expression and soluble MER levels were significantly reduced by Ad Pros1 (Figures S1C,D in Supplementary Material). This confirmed the anti-inflammatory capacity of Ad Pros1. Next, the effect of adenoviral overexpression of *Pros1* in knee joints of mice with KRN STA was examined. In line with the *in vitro* results of *Pros1* overexpression, reduced expression of *Il1b, Tnf*, and *Ccl2* was detected in synovium of mice treated with Ad Pros1 compared to Ad Luc (Figure 2B). Histology taken at day 14 of KRN STA showed a significant reduction of proteoglycan depletion and cartilage erosion in the knee joints of *Pros1*-overexpressing mice (Figures 2C,D). In addition, both bone erosion and the amount of cells infiltrating into the synovium were reduced in mice overexpressing *Pros1* (Figures 2C,D). Consistent with the reduction of cartilage erosion, overexpression of *Pros1* resulted in significantly diminished expression levels of multiple matrix metalloproteinases (MMPs) in the synovium (Figure S2A in Supplementary Material). In constrast to the *in vitro* data, Ad Pros1 did not affect synovial *Adam 17* expression nor the serum concentration of soluble MER (Figures S2B,C in Supplementary Material).

### MER-PROS1 Targeting Modifies the Inflammatory Cytokine Production by Human Three-Dimensional Synovial Micromasses

**Figure 3:**
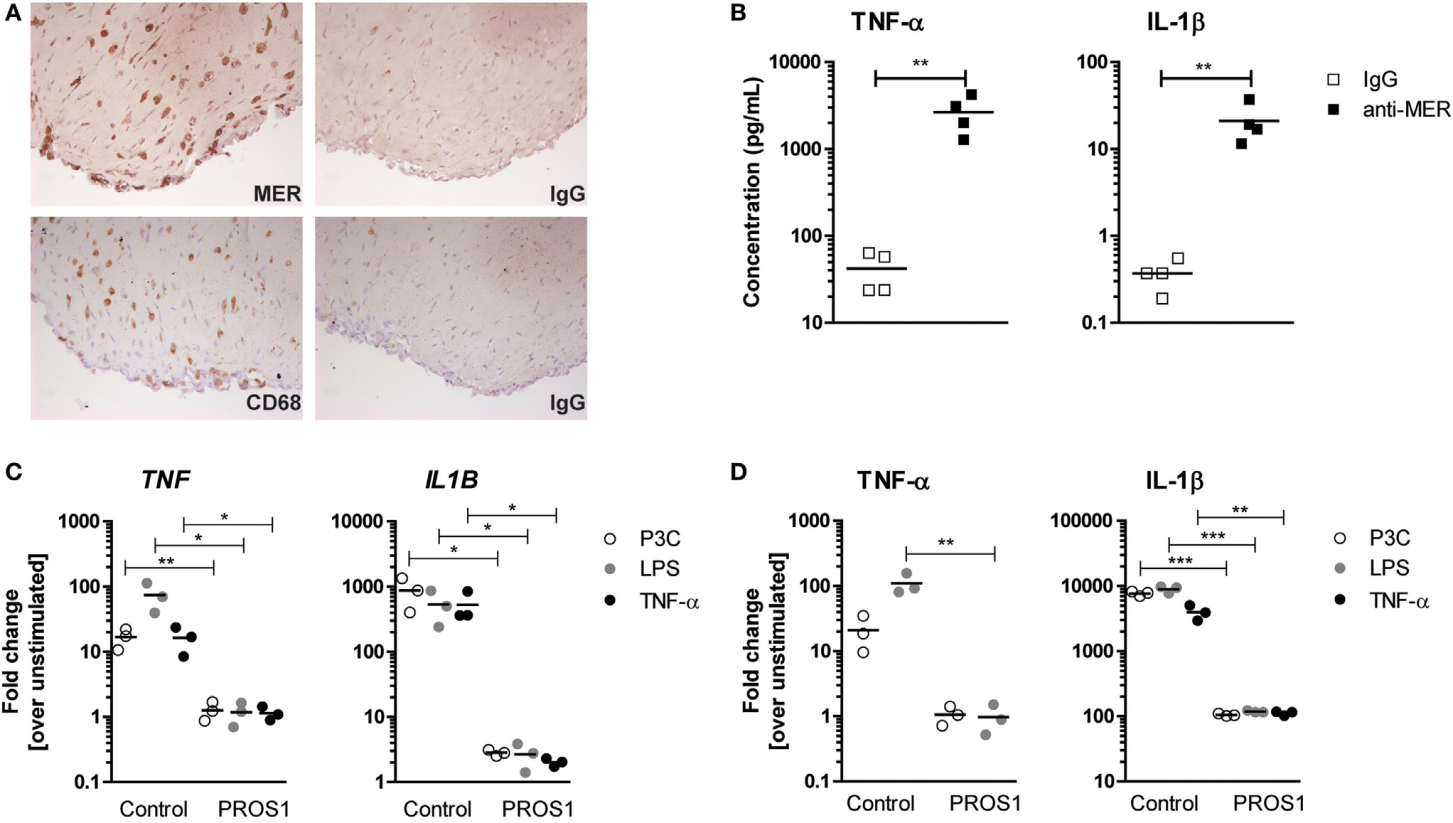
Production of pro-inflammatory cytokines by three-dimensional synovial micromasses after anti-MER or PROS1 treatment. **(A)** Micromasses were examined for their protein expression of MER and CD68 (macrophage marker). Shown are representative pictures and IgG controls. **(B)** Micromasses were treated with IgG or anti-MER for 24 h. Supernatants were examined for the presence of tumor necrosis factor alpha (TNF-α) and IL-1β (*n* = 4). **(C)** Micromasses were preincubated with 50 nM Pros1 for 24 h and stimulated with lipopolysaccharide (LPS) (100 ng/mL), P3C (100 ng/mL), or TNF-α (10 ng/mL) for 6 h. Messenger RNA was extracted and gene expression was determined (*n* = 3). **(D)** Micromasses were pre-incubated with 50 nM Pros1 for 24 h and stimulated with LPS (100 ng/mL), P3C (100 ng/mL), or TNF-α (10 ng/mL) for 24 h. Supernatants were analyzed (*n* = 3). All data are representative for two independent experiments. For **(B–D)**, data are presented as dot-plots with mean. *p < 0.05, **p < 0.01, ***p < 0.001 with unpaired *t*-test. See also Figure S3 in Supplementary Material.

Next, we tested whether MER also mediated an anti-inflammatory response in three-dimensional synovial micromasses consisting out of RAFLS and primary macrophages, a standardized model for the human synovium mimicking the synovial hyperplasia and inflammatory responses observed in RA synovium [manuscript in preparation and published in Ref. (32, 33)]. After 7 days, a lining was formed and immunohistochemistry demonstrated MER^+^ cells dispersed throughout the micromass that had the same morphology as CD68^+^ macrophages (Figure 3A). Without any further stimulation, blocking MER by the addition of MER-specific antibodies significantly enhanced the secretion of TNF-α and IL-1β by human synovial micromasses (Figure 3B). Conversely, addition of PROS1 significantly reduced the gene expression and protein secretion of TNF-α and IL-1β upon stimulation with several TLR ligands or TNF-α (Figures 3C,D). None of the conditions influenced the expression of *ADAM17* or the shedding of MER (Figure S3 in Supplementary Material). This showed that MER exerted an anti-inflammatory effect under naive conditions that could be further enhanced by the addition of PROS1.

### MER-Specific Antibodies Aggravate Arthritis Pathology

**Figure 4:**
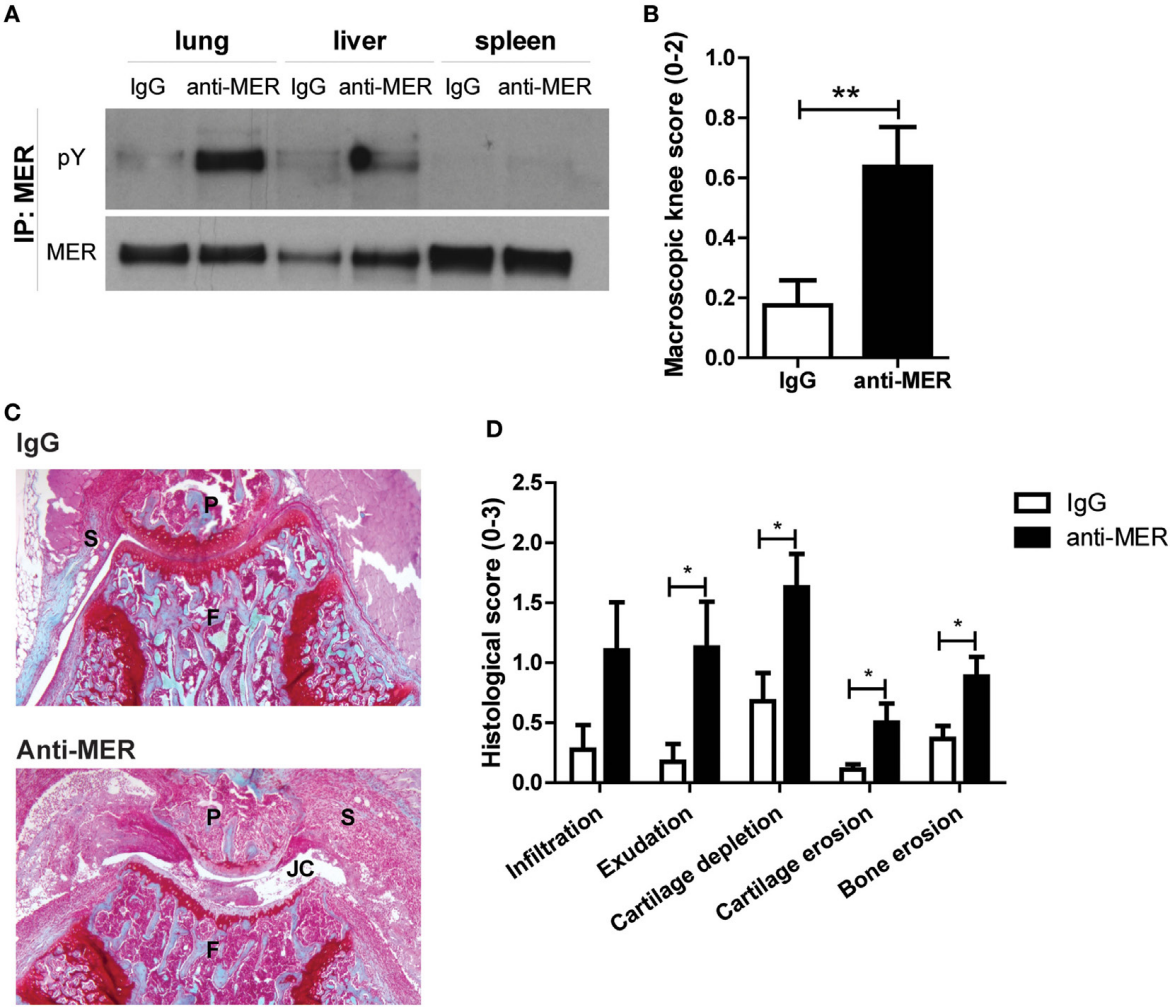
Effect of MER-specific antibody treatment on clinical severity and pathology of collagen-induced arthritis (CIA). **(A)** Mice with CIA were intravenously injected with IgG or anti-MER (*n* = 11–12 mice). Two mice were euthanized 1 h after injection. Lung, liver, and spleen were isolated and samples were immunoprecipitated (IP) for MER and immunoblotted for MER or phosphorylation (pY) (*n* = 1). **(B)** Macroscopic score of knee joints as determined by swelling and blood vessel formation (*n* = 20–22 knee joints). **(C)** Shown are representative pictures of histological knee joint sections stained with safranin O and fast green of each group. **(D)** Histological knee joint sections were scored for the arthritis parameters depicted. Three semi-serial sections per joint were scored in a random and blinded manner with an arbitrary score from 0 to 3 (*n* = 20–22 knee joints). P, patella; F, femur; S, synovium; JC, joint cavity. For **(B–D)**, data are presented as mean + SEM. *p < 0.05, **p < 0.01 with unpaired *t*-test. See also Figure S4 in Supplementary Material.

Treatment of RA patients with PROS1 will not be the first choice because the majority of PROS1 complexes with C4b-binding protein making it inactive and it only has a half-life of approximately 2 days (34–37). Moreover, free PROS1 contains potent anti-coagulation activity (38). A more efficient and safer approach would be the use of MER-specific antibodies that were previously described to activate MER kinase activity (21, 31). We tested this approach in the CIA model. Anti-MER or IgG was administered intravenously at day 22 of CIA and this led to MER phosphorylation in lung and to a lesser extent liver 1 h thereafter (Figure 4A). Unexpectedly, anti-MER treatment markedly increased arthritis severity, determined by macroscopic knee swelling and blood vessel formation at day 30 of CIA (Figure 4B). Moreover, histology of knee joints showed that the anti-MER treatment resulted in a trend toward more infiltrating cells in the synovial lining (infiltrate) and significant higher amount of cells in the joint cavity (exudate). Furthermore, higher cartilage depletion, and cartilage and bone erosion were observed after administration of anti-MER (Figures 4C,D). To determine the effect of anti-MER on the local expression of cytokines and MMPs, gene expression analysis of synovium was performed. Consistent with the increased bone destruction, enhanced expression of *Il17a* was observed in synovium of mice that received anti-MER. A trend of enhanced expression of *Il6* and *Cxcl1* in synovium and IL-6 and KC protein levels in sera was observed after anti-MER treatment (Figures S4A,B in Supplementary Material). Moreover, anti-MER significantly enhanced *Mmp9* and *Mmp13* expression in the synovium, consistent with increased cartilage erosion (Figure S4C in Supplementary Material). The enhanced synovial *Socs3* expression could be an indication that the MER-specific antibodies evoked a local anti-inflammatory response (Figure S4D in Supplementary Material), but if so, it appeared to be overruled during arthritis. Anti-MER did neither induce shedding of soluble MER in the serum nor did it alter *Adam17* expression (Figures S4E,F in Supplementary Material).

### MER Mediates Efferocytosis in the Arthritic Knee Joint

**Figure 5:**
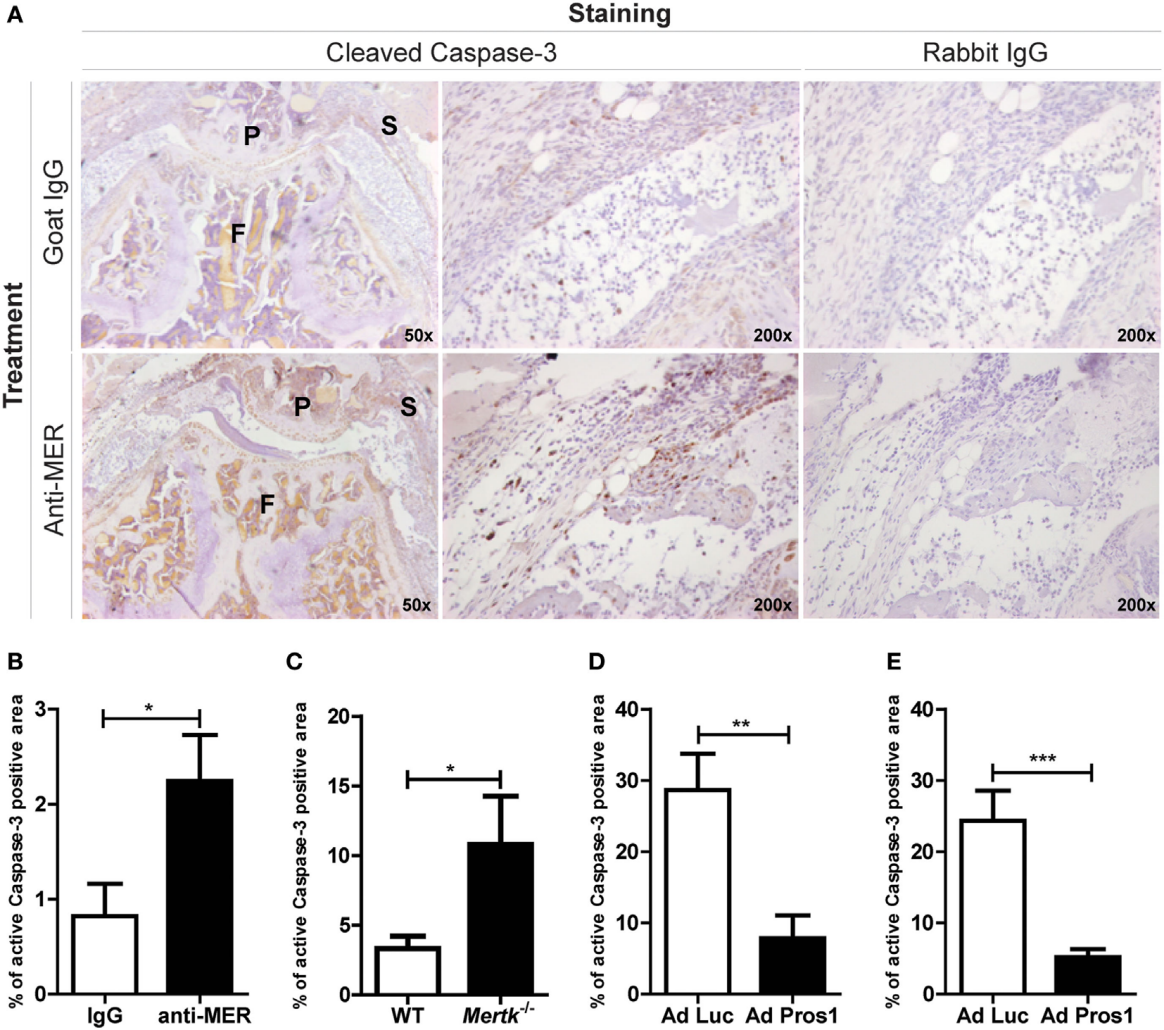
Evaluating the outcome on efferocytosis after treatment with MER-specific antibodies, *Mertk* gene ablation or *Pros1* overexpression. **(A)** Immunohistochemical staining for cleaved Caspase-3 in knee joints of collagen-induced arthritis (CIA) mice injected with IgG or anti-MER. Shown are representative pictures of knee joint sections with similar microscopic scores of inflammation, in two different magnifications. **(B)** Pictures of knee joint sections shown in **(A)** were quantified and the cleaved Caspase-3 positive area was corrected by the total inflamed area (*n* = 10–12 knee joints). **(C)** Quantification of immunohistochemical staining for cleaved Caspase-3 in knee joints of wild-type (WT) or *Mertk^−/−^* mice with KRN serum transfer arthritis (STA) mice (*n* = 10–18 knee joints). **(D)** Quantification of immunohistochemical staining for cleaved Caspase-3 in knee joints of CIA mice intravenously injected with Ad Luc or Ad Pros1 (*n* = 18 knee joints). **(E)** Quantification of immunohistochemical staining for cleaved Caspase-3 in knee joints of KRN STA mice intra-articularly injected with Ad Luc or Ad Pros1 (*n* = 12 knee joints). P, patella; F, femur; S, synovium. For **(B–E)**, data are presented as mean + SEM. *p < 0.05, **p < 0.01, ***p < 0.001 with unpaired *t*-test. See also Figure S5 in Supplementary Material.

Administration of MER-specific antibodies in mice with CIA enhanced the joint inflammation and more apoptotic cells were present as detected by immunohistochemical staining of cleaved Caspas-3 (Figure 5A), a marker for apoptotic cells (7). Quantitative image analysis showed that the cleaved Caspase-3 positive area within the inflamed area was increased by anti-MER treatment (Figure 5B). This suggests that the MER-specific antibodies could have negatively influenced efferocytosis during arthritis in the knee joint. In agreement with this, anti-MER reduced the efferocytosis of apoptotic cells by J774A.1 macrophages in a dose-dependent manner (Figure S5A in Supplementary Material). To confirm the role of MER in efferocytosis during arthritis, knee joint sections from previous *in vivo* experiments were stained for cleaved Caspase-3 and quantified. In line with the results of the anti-MER experiments, more apoptotic cells in the knee joints of *Mertk^−/−^* mice with KRN STA were detected (Figure 5C). Systemic adenoviral overexpression of the MER ligand PROS1 showed less apoptotic cells in the KNR STA model (Figure 5D) and CIA model (Figure 5E), indicating efferocytosis was enhanced by *Pros1* overexpression. Altogether, these results show that MER is involved in efferocytosis in the arthritic knee joint and that MER-specific antibodies inhibited this process.

### MER-Mediated Efferocytosis Limits the Secondary Necrosis of Apoptotic Cells in the Arthritic Joint

**Figure 6:**
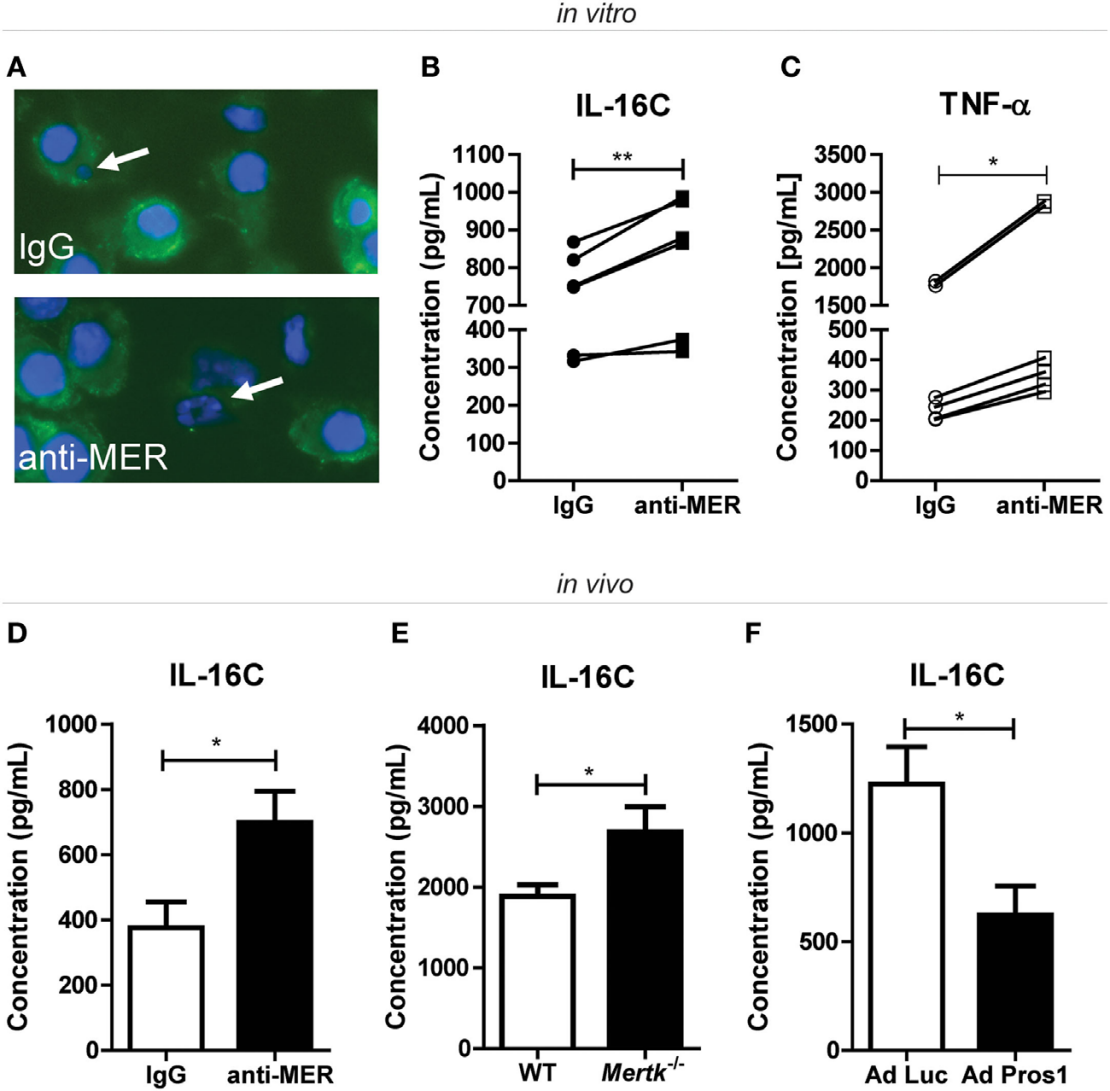
Assessment of secondary necrosis after treatment with MER-specific antibodies, *Mertk* gene ablation or *Pros1* overexpression. **(A)** J774A.1 cells were incubated with IgG or anti-MER and cocultured with bone marrow neutrophils for 24 h. Cells were stained with F4/80 (green) and DAPI (blue). Representative pictures are shown for each group. White arrows indicate an engulfed cell (IgG group) or an apoptotic cell (anti-MER group). **(B)** J774A.1 cells were incubated with IgG or anti-MER and cocultured with bone marrow neutrophils for 48 h. Supernatants were examined for the presence of interleukin (IL)-16C (*n* = 6 individual experiments). **(C)** Supernatants of **(B)** were analyzed for the presence of tumor necrosis factor alpha (TNF-α) (*n* = 6 individual experiments). **(D)** Serum samples from collagen-induced arthritis (CIA) mice systemically treated with IgG or anti-MER were analyzed for IL-16C (*n* = 10–11 mice). **(E)** Serum samples from wild-type (WT) or *Mertk^−/−^* mice with KRN serum transfer arthritis were analyzed for IL-16C (*n* = 9–15 mice). **(F)** Serum samples from mice with CIA intravenously injected with Ad Luc or Ad Pros1 were analyzed for IL-16C (*n* = 7–8 mice). For **(B,C)**, data are presented as dot-plots. *p < 0.05, **p < 0.01 with paired *t*-test. For **(D–F)**, data are presented as mean + SEM. *p < 0.05 with unpaired *t*-test. See also Figure S5 in Supplementary Material.

Effective clearance of apoptotic cells by efferocytosis is essential for tissue homeostasis. If this process is impaired, apoptotic cells go into a process of secondary necrosis (39, 40). When neutrophils go into secondary necrosis, the intracellular inactive pre-IL-16 is cleaved in a Caspase-3-dependent manner to the biologically active IL-16C and released (40). To show that blocking MER-mediated efferocytosis leads to secondary necrosis, neutrophils were cocultured with macrophages in the presence of anti-MER or IgG. Anti-MER prevented the uptake of apoptotic neutrophils (Figure 6A) and culture supernatants showed increased IL-16C (Figure 6B) and TNF-α levels (Figure 6C). The IL-16C most likely originated from neutrophils, as IL-16C was not detectable in this assay in the absence of neutrophils. In these conditions, TNF-α protein levels and *Adam17* expression were unaltered. However, anti-MER did abolish all soluble MER in the supernatant (Figures S5B–D in Supplementary Material). Anti-MER administration led to a significant increase of systemic IL-16C protein levels at day 30 of CIA (Figure 6D), suggesting that prevention of efferocytosis by blocking MER led to secondary necrosis of neutrophils in the arthritic joint. In addition, systemic IL-16C levels were significantly higher in *Mertk*-deficient mice (Figure 6E). Conversely, systemic levels of IL-16C were significantly reduced in CIA mice treated with Ad Pros1 compared to Ad Luc (Figure 6F). This showed that MER plays a protective role during arthritis by mediating efferocytosis and that this nullifies the protective effect of agonistic MER-specific antibodies in arthritis. Blocking of efferocytosis by anti-MER led to a pro-inflammatory cascade initiated by the secondary necrosis of apoptotic neutrophils.

## Discussion

Our study shows that MER plays a protective role during experimental arthritis in the macrophage-dependent KRN STA model (41), the T and B cell-dependent CIA model (42, 43), and in a three-dimensional model of the human synovium. We found that activating MER by PROS1 is indeed anti-inflammatory and also ameliorates arthritis. However, this could not be mimicked by agonistic MER-specific antibodies, which also possess the bivalent function of blocking MER-mediated efferocytosis.

Mice with a deficiency of *Mertk* showed aggravated diseases and treatment with *Pros1* by viral overexpression ameliorated the disease in the KRN STA model. The latter confirmed our previously published observation of the protective effect of *Pros1* overexpression in the CIA model (16). MER activation *via* PROS1 delivers a negative feedback signal *via* the induction of SOCS3 that dampens inflammatory cytokine response (10, 11, 13–17). Indeed, we found increased synovial expression of this gene in the CIA model after *Pros1* overexpression (16). This could explain the arthritis protective effect of MER activation in the mouse models and anti-inflammatory effect of MER activation in the human synovial micromass model. It is also in line with more severe arthritis observed in *Mertk*-deficient mice and higher cytokine release by human synovial micromasses in the presence of blocking MER-specific antibodies. Whether this is all due to SOCS3 induction or through inducing specialized proresolving mediators (21), remains to be determined.

Based on our results that the endogenous protective role of MER during arthritis could be further enhanced by exogenously delivered PROS1 or viral overexpression, we envision that MER targeting would be a therapeutic option to treat RA patients. However, PROS1 will not be the first choice because this protein has a half-life of only 2 days and its availability is reduced due to complex formation with C4b-binding protein (34–37). Local viral delivery of the *Pros1* gene could be an option, but safety is still an issue and an unlikely strategy to treat all affected joints in the RA patient. For that, treatment of RA patients with an agonistic MER-specific antibody would be safer and possibly also more efficient. This was tested in the CIA model and unexpectedly, we found that this specific anti-MER antibody treatment deteriorated the disease due to accumulation of apoptotic cells in the joint that go into secondary necrosis and release their pro-inflammatory content thereby fueling joint inflammation. Although we cannot exclude that anti-MER suboptimally activates the MER receptor, we can conclude that anti-MER interferes with MER-mediated efferocytosis and appears to have a bivalent function: activation of the receptor but also blocking of MER-mediated efferocytosis. It does not appear that the inhibition of efferocytosis is due to shedding of the MER receptor. In all *in vivo* studies, serum levels of soluble MER and *Adam17* expression were unaltered. *In vitro*, anti-MER and adenoviral *Pros1* completely abolished the shedding of MER. The complete absence of soluble MER by MER activation could be due to the internalization of the receptor upon activation, a common method deployed by receptor tyrosine kinases (44).

The importance of MER in efferocytosis is demonstrated by the fact that adult *Mertk^−/−^* mice exhibit apoptotic cell accumulation in multiple tissues (13, 45, 46). During arthritis, neutrophils migrate in high numbers into the joint cavity (47). Neutrophils are short-lived cells and die by apoptosis (48). Apoptotic cells which are not taken up by phagocytes undergo secondary necrosis (39, 40, 49). We showed that neutrophils (which do not express *Mertk*) went into secondary necrosis when cocultured with anti-MER-treated macrophages, as shown by increased levels of IL-16C. IL-16C is processed from its inactive pre-form in a Caspase-3-dependent manner to the biologically active IL-16C, in neutrophils (40). Additionally, serum of anti-MER-treated mice and *Mertk^−/−^* mice contained higher levels of IL-16C during arthritis. Conversely, protective effects were observed when *Pros1* was virally overexpressed as IL-16C serum levels were reduced in these mice. IL-16C is a pro-inflammatory cytokine that induces among other IL-6 (50). We found that the IL-16C levels correlated significantly with the synovial gene expression of *Il6* (*R*^2^ = 0.8349; *p* = 0.0002) in the CIA model. Enhanced levels of IL-16 are observed in RA patients, associated with joint destruction and inflammation, making it tempting to speculate secondary necrosis plays an aggravating role during RA (51, 52). Noteworthy, IL-16 is an extraordinarily effective parameter to measure clinical responses during early treatment (53).

Little has been described about the TAM receptors and their ligands in human synovium. One study showed that human RA fibroblast-like synoviocytes respond to GAS6, likely *via* TYRO3 and AXL (54). Another study showed the presence of AXL in human synovial lining cells and also the presence of GAS6 in synovium and synovial fluid (55). We showed that targeting the MER-PROS1 axis in a human three-dimensional model of the synovium is beneficial both at homeostasis and in an inflammatory environment. Moreover, the cleaved soluble form of MER, present in human synovial fluid of RA patients, impairs efferocytosis (manuscript in preparation).

PROS1 protein therapy and *PROS1* gene therapy are not the first choice for treatment of RA patients whereas the use of antibodies, such as anti-TNF-α, is the first-line biological after methotrexate. However, our study showed that activating the MER receptor using an antibody strategy may have a counterproductive effect of blocking MER-mediated efferocytosis, leading to necrotic cell death that stimulates inflammation. In summary, our data show that MER-mediated efferocytosis plays a crucial role in the arthritis process suggesting that preserving or stimulating this pathway is a therapeutic option in RA.

## Ethics Statement

RA synovial tissue was obtained during surgery from the Radboud University Medical Center or the Sint Maartenskliniek (both the Netherlands). This material was considered surgery surplus material. For the Radboud University Medical Center, patients gave informed consent for the surgery, were informed and were able to decline the use of their material for research. According to Dutch law, informed consent was not necessary. In addition, patient material was anonymized. For the Sint Maartenskliniek, patients gave written informed consent for the use of their material for research. The patient material was pseudonymized. Therefore, there was no need for the approval by an ethical committee. Protocols were performed in accordance to the code of conduct for responsible use of human tissue in medical research (Gedragscode 2011, https://www.federa.org/code-goed-gebruik). For animal subjects: All *in vivo* studies performed in The Netherlands complied with Dutch legislation and were approved by local authorities for the care and use of animals with related codes of practice. The *in vivo* studies executed in The United States of America were conducted according to guidelines established by the Salk Institutional Animal Care and Use Committee.

## Author Contributions

Conceptualization and methodology: CW, SB, MGAB, and FL; validation: CW, SB, and MGAB; formal analysis: CW and SB; investigation: CW, SB, MGAB, and MBB; resources: MK; writing—original draft: CW, SB, and FL; writing—review and editing: MGAB, MBB, MK, PL, WB, and PK; visualization: CW; supervision: FL; funding acquisition: SB and FL.

## Conflict of Interest Statement

The authors declare that the research was conducted in the absence of any commercial or financial relationships that could be construed as a potential conflict of interest.

## Supplementary Material

Figure S1 Expression of MER, soluble MER and, Pros1and Adam17 by bone marrow-derived macrophages (BMMs). **(A)** BMMs were analyzed with flow cytometry for the membrane protein expression of MER. Red = unstained, blue = stained for MER. **(B,C)** BMMs were transduced with Ad Luc or Ad Pros1 and stimulated with lipopolysaccharide (LPS) (100 ng/mL) or P3C (100 ng/mL) for 6 h. Messenger RNA was extracted and gene expression was determined (*n* = 3 per experiment). **(D)** BMMs were transduced with Ad Luc or Ad Pros1 and stimulated with LPS (100 ng/mL) or P3C (100 ng/mL) for 24 h. Supernatants were analyzed (*n* = 3 per experiment). For **(B)**, data are presented as dot-plots with mean. For **(C,D)**, data are presented as mean + SEM. **p* < 0.01, ****p* < 0.001 with unpaired *t*-test comparing Ad Luc to Ad Pros1 conditions. All data are representative for two independent experiments. ND = not detected. See also Figure 2.

Figure S2 Effect of local adenoviral Pros1 overexpression on metalloproteinase expression, Adam17 expression and soluble MER levels in KRN STA. KRN STA was induced in mice overexpressing luciferase (Ad Luc) or Pros1 (Ad Pros1) in their knee joints and mice were euthanized at day 14. **(A,B)** Knee synovial biopsies were obtained, mRNA was extracted and gene expression was determined (*n* = 6 knee synovial biopsies). **(C)** Serum samples from the same mice were analyzed for soluble MER (*n* = 12 mice). Data are represented as mean + SEM. ***p* < 0.01, ****p* < 0.001 with unpaired *t*-test. See also Figure 2.

Figure S3 Expression of Pros1, Adam17, and soluble MER levels by three-24 dimensional synovial micromasses after anti-MER or PROS1 treatment. **(A–C)** Micromasses were treated with IgG or anti-MER for 24 h, mRNA was extracted and gene expression was determined (*n* = 4). (B) Micromasses were treated with IgG or anti-MER for 24 h. Supernatants were examined for the presence of soluble MER (*n* = 4). **(D,E)** Micromasses were preincubated with 50 nM Pros1 for 24 h and stimulated with lipopolysaccharide (LPS) (100 ng/mL), P3C (100 ng/mL) or tumor necrosis factor alpha (TNF-α) (10 ng/mL) for 6 h. Messenger RNA was extracted, and gene expression was determined (*n* = 3). **(F)** Micromasses were pre-incubated with 50 nM Pros1 for 24 h and stimulated with LPS (100 ng/mL), P3C (100 ng/mL) or TNF-α (10 ng/mL) for 24 h. Supernatants were analyzed (*n* = 3). All data are representative for two independent experiments. For **(A–C)**, data are presented as dot-plots with mean. For **(D–F)**, data are presented as mean + SEM. See also Figure 3.

Figure S4 Local and systemic effects of anti-MER on inflammation and destruction in collagen-induced arthritis (CIA). Knee synovial biopsies were obtained at day 30 from mice with CIA intravenously injected with 10 µg IgG or anti-MER. Messenger RNA was extracted and gene expression was determined for **(A)** cytokines and chemokines, **(C)** metalloproteinases, or **(D)** SOCS genes and **(E)** Adam17 (*n* = 10 knee synovial biopsies). **(B,F)** Serum samples from the same mice were analyzed for IL-6 and KC by Bio-Plex Multiplex Immunoassay or soluble MER (*n* = 10–11 mice). All data are presented as mean + SEM. **p* < 0.05, ***p* < 0.01 with unpaired *t*-test. See also Figure 4.

Figure S5 Effect of anti-MER on efferocytosis. **(A)** J774A.1 cells were incubated with IgG or anti-MER and cocultured with pHrodo-labeled apoptotic cells. Efferocytosis was analyzed by fluorescence microscopy (*n* = 6). **(B)** J774A.1 cells were incubated with IgG or anti-MER, mRNA was extracted and gene expression was determined (*n* = 3–4). **(C)** J774A.1 cells were incubated with IgG or anti-MER. Supernatants were examined for the presence of soluble MER (*n* = 4). **(D)** J774A.1 cells were incubated with IgG or anti-MER. Supernatants were examined for the presence of soluble MER (*n* = 4). For **(A)**, data are presented as mean + SD. For **(B–D)**, data are presented as dot-plots with mean. For **(A)**, ***p* < 0.01 with one-way ANOVA with Bonferroni post test. ND, not detected. See also Figure 5 and 6.
