# Supplementary figures and images for: Protective Role of the MER Tyrosine Kinase via Efferocytosis in Rheumatoid Arthritis Models
Source: Front Immunol. 2018 Apr 13;9:742. doi: 10.3389/fimmu.2018.00742 (PMC5908888; doi:10.3389/fimmu.2018.00742)

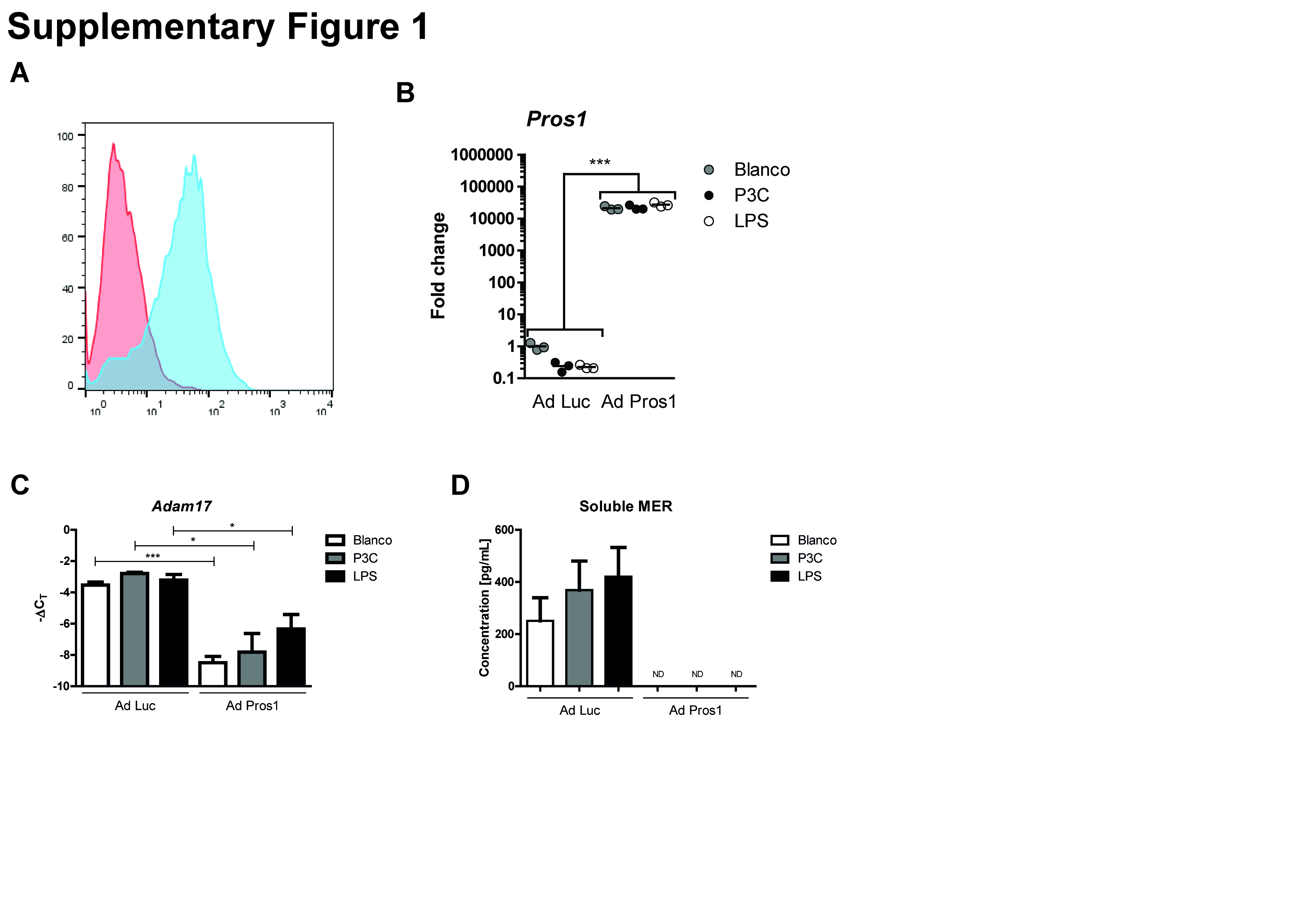

Supplement: Figure S1 — Expression of MER, soluble MER and, Pros1and Adam17 by bone marrow-derived macrophages (BMMs). (A) BMMs were analyzed with flow cytometry for the membrane protein expression of MER. Red = unstained, blue = stained for MER. (B,C) BMMs were transduced with Ad Luc or Ad Pros1 and stimulated with lipopolysaccharide (LPS) (100 ng/mL) or P3C (100 ng/mL) for 6 h. Messenger RNA was extracted and gene expression was determined (n = 3 per experiment). (D) BMMs were transduced with Ad Luc or Ad Pros1 and stimulated with LPS (100 ng/mL) or P3C (100 ng/mL) for 24 h. Supernatants were analyzed (n = 3 per experiment). For (B), data are presented as dot-plots with mean. For (C,D), data are presented as mean + SEM. *p < 0.01, ***p < 0.001 with unpaired t-test comparing Ad Luc to Ad Pros1 conditions. All data are representative for two independent experiments. ND = not detected. See also Figure 2. [file Image_1.tif]

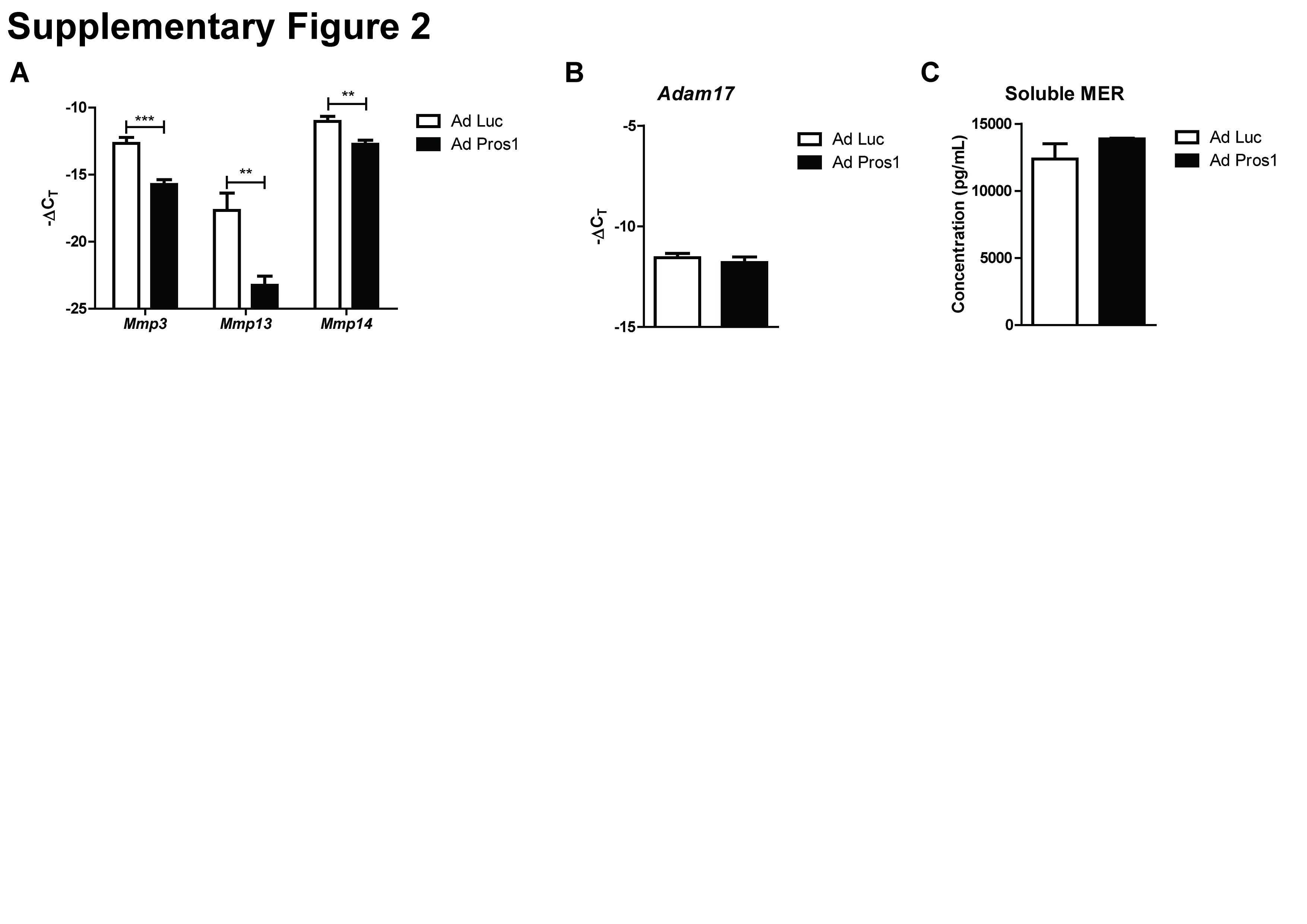

Supplement: Figure S2 — Effect of local adenoviral Pros1 overexpression on metalloproteinase expression, Adam17 expression and soluble MER levels in KRN STA. KRN STA was induced in mice overexpressing luciferase (Ad Luc) or Pros1 (Ad Pros1) in their knee joints and mice were euthanized at day 14. (A,B) Knee synovial biopsies were obtained, mRNA was extracted and gene expression was determined (n = 6 knee synovial biopsies). (C) Serum samples from the same mice were analyzed for soluble MER (n = 12 mice). Data are represented as mean + SEM. **p < 0.01, ***p < 0.001 with unpaired t-test. See also Figure 2. [file Image_2.tif]

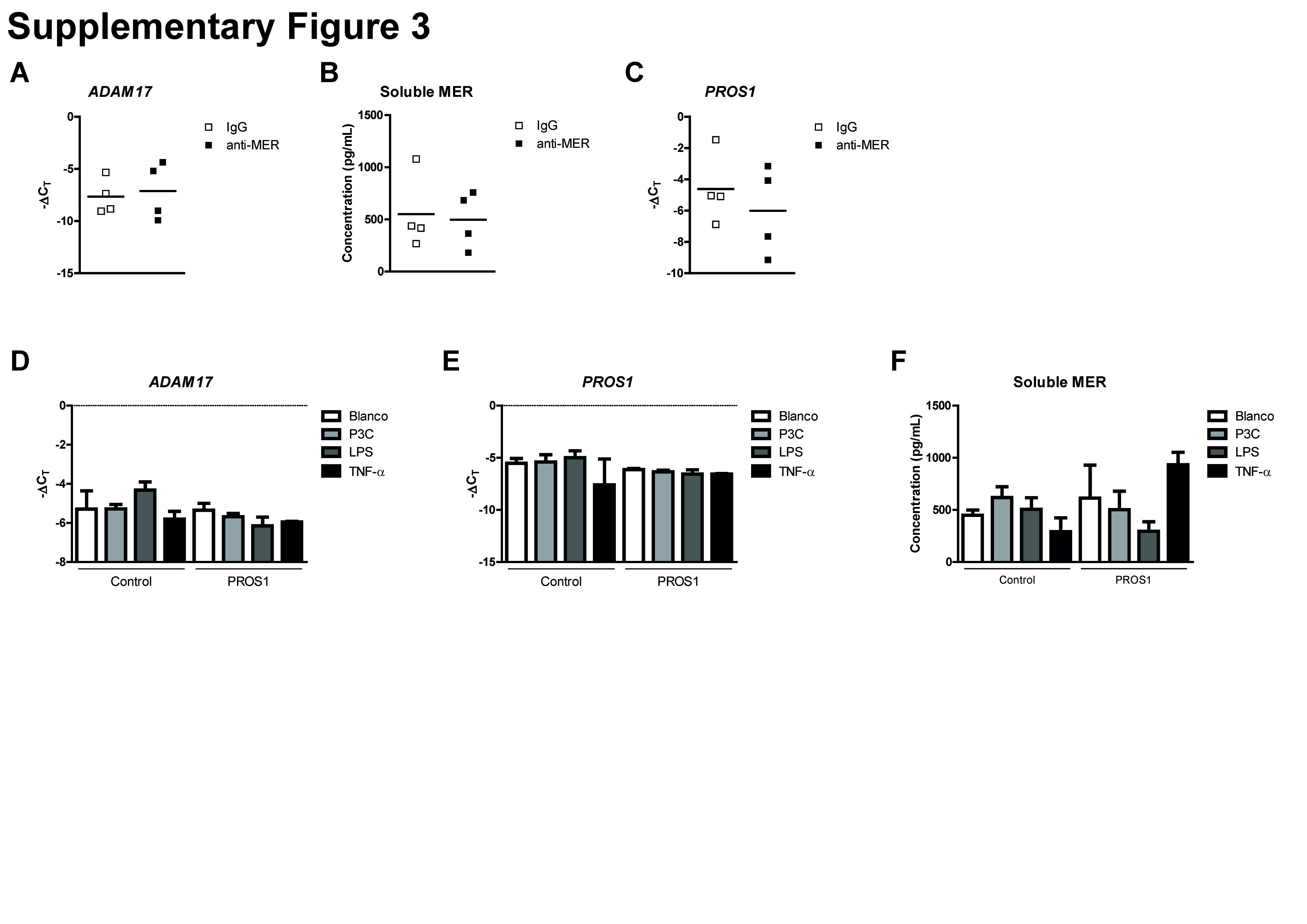

Supplement: Figure S3 — Expression of Pros1, Adam17, and soluble MER levels by three-24 dimensional synovial micromasses after anti-MER or PROS1 treatment. (A–C) Micromasses were treated with IgG or anti-MER for 24 h, mRNA was extracted and gene expression was determined (n = 4). (B) Micromasses were treated with IgG or anti-MER for 24 h. Supernatants were examined for the presence of soluble MER (n = 4). (D,E) Micromasses were preincubated with 50 nM Pros1 for 24 h and stimulated with lipopolysaccharide (LPS) (100 ng/mL), P3C (100 ng/mL) or tumor necrosis factor alpha (TNF-α) (10 ng/mL) for 6 h. Messenger RNA was extracted, and gene expression was determined (n = 3). (F) Micromasses were pre-incubated with 50 nM Pros1 for 24 h and stimulated with LPS (100 ng/mL), P3C (100 ng/mL) or TNF-α (10 ng/mL) for 24 h. Supernatants were analyzed (n = 3). All data are representative for two independent experiments. For (A–C), data are presented as dot-plots with mean. For (D–F), data are presented as mean + SEM. See also Figure 3. [file Image_3.tif]

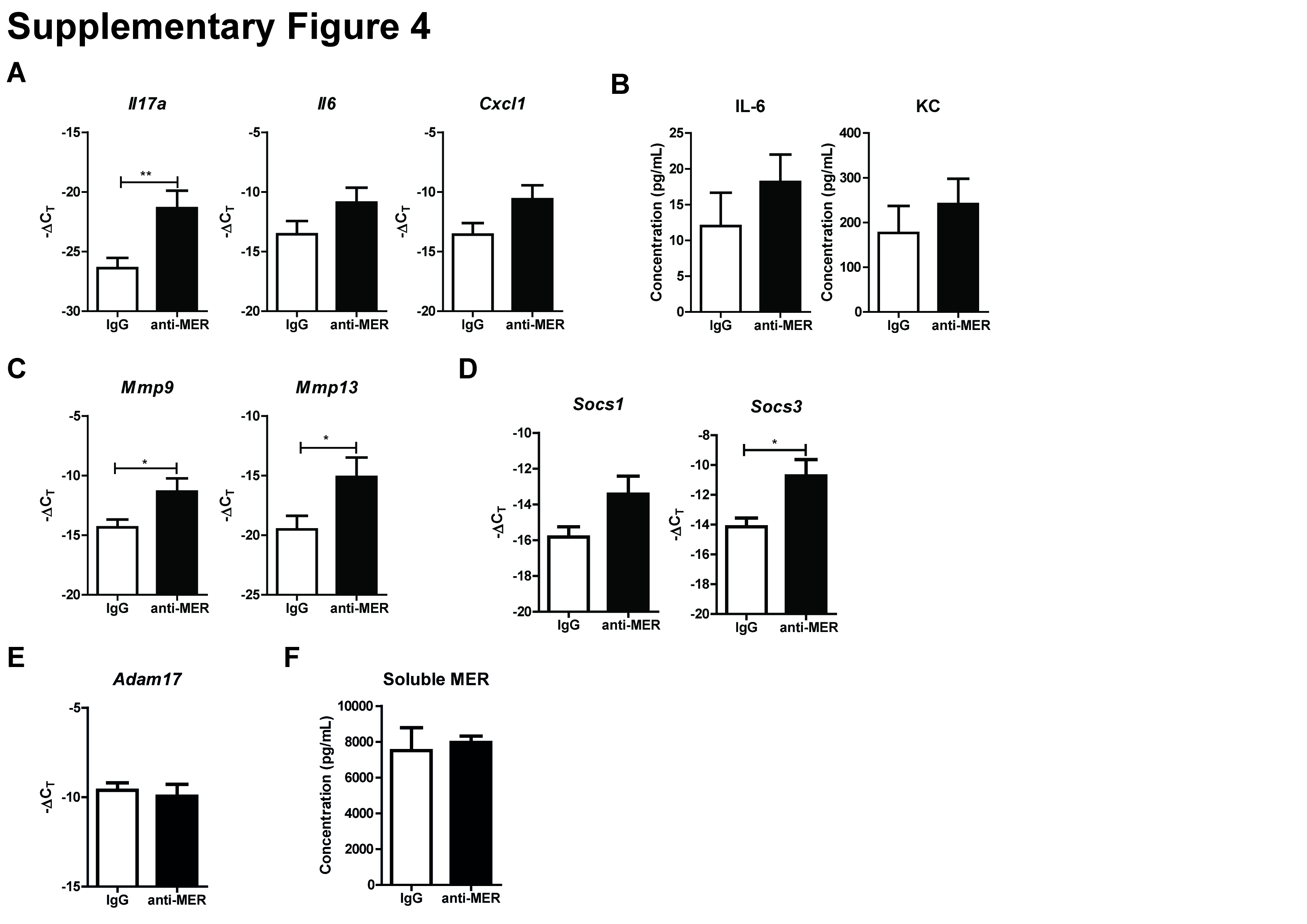

Supplement: Figure S4 — Local and systemic effects of anti-MER on inflammation and destruction in collagen-induced arthritis (CIA). Knee synovial biopsies were obtained at day 30 from mice with CIA intravenously injected with 10 µg IgG or anti-MER. Messenger RNA was extracted and gene expression was determined for (A) cytokines and chemokines, (C) metalloproteinases, or (D) SOCS genes and (E) Adam17 (n = 10 knee synovial biopsies). (B,F) Serum samples from the same mice were analyzed for IL-6 and KC by Bio-Plex Multiplex Immunoassay or soluble MER (n = 10–11 mice). All data are presented as mean + SEM. *p < 0.05, **p < 0.01 with unpaired t-test. See also Figure 4. [file Image_4.tif]

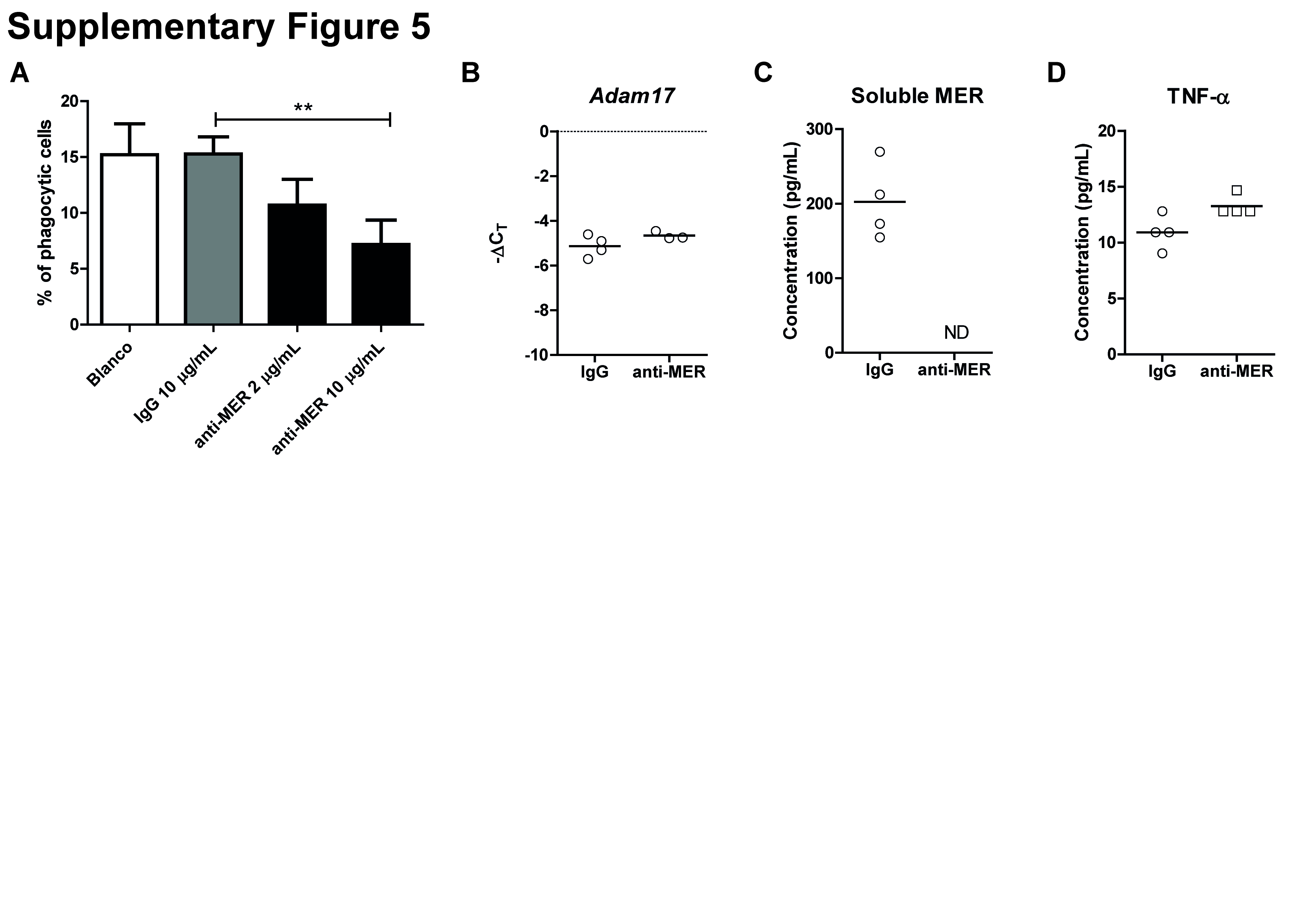

Supplement: Figure S5 — Effect of anti-MER on efferocytosis. (A) J774A.1 cells were incubated with IgG or anti-MER and cocultured with pHrodo-labeled apoptotic cells. Efferocytosis was analyzed by fluorescence microscopy (n = 6). (B) J774A.1 cells were incubated with IgG or anti-MER, mRNA was extracted and gene expression was determined (n = 3–4). (C) J774A.1 cells were incubated with IgG or anti-MER. Supernatants were examined for the presence of soluble MER (n = 4). (D) J774A.1 cells were incubated with IgG or anti-MER. Supernatants were examined for the presence of soluble MER (n = 4). For (A), data are presented as mean + SD. For (B–D), data are presented as dot-plots with mean. For (A), **p < 0.01 with one-way ANOVA with Bonferroni post test. ND, not detected. See also Figure 5 and 6. [file Image_5.tif]
